# Developmental Reading-Network Reorganization in Developmental Dyslexia: A Neuroplasticity Framework

**DOI:** 10.3390/brainsci16080833

**Published:** 2026-08-06

**Authors:** Sujood Kitany, Salim Abu-Rabia, Rami Arfaiya

**Affiliations:** 1 Department of Special Education, Faculty of Education, Haifa University, Haifa 3498838, Israel; so.m.masre@gmail.com (S.K.); rami0407@gmail.com (R.A.); 2 The Academic Arabic College in Israel-Haifa, Haifa 33145, Israel

**Keywords:** developmental dyslexia, reading network, neuroplasticity, right hemisphere, reading acquisition, brain connectivity, white matter, developmental neuroscience, longitudinal neuroimaging, network reorganization

## Abstract

**Highlights:**

**What are the main findings?**

- A developmental neuroplasticity framework is proposed to integrate evidence on reading-network reorganization in developmental dyslexia.
- Early right-hemisphere recruitment is interpreted as one possible manifestation of developmental reading-network reorganization rather than a universal compensatory mechanism.

**What are the implications of the main findings?**

- The framework generates testable hypotheses for future longitudinal and intervention studies.
- A developmental perspective may provide a framework for interpreting heterogeneous neurobiological findings and support more developmentally informed approaches to reading intervention.

**Abstract:**

Developmental dyslexia is a common neurodevelopmental disorder characterized by persistent difficulties in accurate and fluent word reading despite adequate intelligence, educational opportunity, and intact sensory function. Contemporary neurobiological models have progressively shifted from localization-based explanations toward network-oriented perspectives emphasizing large-scale brain connectivity, developmental maturation, and neuroplasticity. Nevertheless, the developmental mechanisms underlying early right-hemisphere recruitment remain incompletely understood. Although increased right-hemisphere activation has traditionally been interpreted as a compensatory response to left-hemisphere dysfunction, accumulating evidence from longitudinal neuroimaging, intervention, and developmental studies indicates that this explanation alone does not adequately account for the heterogeneity of neurobiological findings observed across individuals with developmental dyslexia. This narrative review synthesizes evidence from developmental neurobiology, network neuroscience, longitudinal neuroimaging, and intervention research to examine the biological processes underlying early right-hemisphere recruitment. Across the reviewed literature, developmental dyslexia is increasingly characterized by atypical maturation of distributed reading networks involving alterations in white-matter development, functional connectivity, hemispheric lateralization, and experience-dependent neuroplasticity. Collectively, these findings suggest that reading networks remain dynamically modifiable throughout literacy acquisition and that multiple developmental pathways may contribute to diverse neurobiological and behavioral outcomes. Building on this evidence, we propose a Developmental Neuroplasticity Framework for Reading Network Reorganization that integrates existing neurobiological models within a unified developmental perspective. Rather than proposing a new neurobiological mechanism, the framework seeks to explain the heterogeneous patterns of early right-hemisphere recruitment that are not fully accounted for by compensation-based interpretations alone. Specifically, it conceptualizes early right-hemisphere recruitment as one possible adaptive developmental outcome emerging from interactions among early neurodevelopmental vulnerability, distributed network connectivity, developmental neuroplasticity, and environmental experience. By integrating evidence that has largely been considered within separate theoretical perspectives, the framework generates empirically testable predictions regarding developmental trajectories, network reorganization, and intervention-related variability, while providing a conceptual basis for earlier identification of children at risk and the development of more targeted, developmentally informed intervention strategies.

## 1. Introduction

Developmental dyslexia is among the most common neurodevelopmental disorders, affecting approximately 5–10% of school-aged children and representing a major cause of persistent difficulties in accurate and fluent word reading despite adequate intelligence, educational opportunity, and intact sensory functioning [1]. Over the past three decades, advances in cognitive neuroscience and developmental neuroimaging have substantially expanded our understanding of the neural mechanisms underlying reading acquisition. Rather than depending on a single cortical region, successful reading is now recognized as the product of coordinated interactions among distributed neural systems whose structural and functional organization continues to mature throughout childhood [2].

Early neurobiological models primarily emphasized dysfunction within left-hemisphere language regions as the principal neural basis of developmental dyslexia. More recent research, however, has shifted toward a network-based perspective, characterizing reading as a dynamic neurodevelopmental process supported by interactions among cortical regions, white-matter pathways, and large-scale functional connectivity [3]. Longitudinal neuroimaging studies further demonstrate that many neural differences associated with developmental dyslexia emerge before formal reading instruction and continue to evolve throughout literacy acquisition, highlighting the importance of developmental processes rather than static neural abnormalities [4]. Consequently, contemporary research increasingly emphasizes distributed network organization, brain connectivity, and developmental neuroplasticity as central features of reading development.

Within this developmental perspective, growing attention has focused on evidence that some children and adults with developmental dyslexia recruit right-hemisphere regions during reading to a greater extent than typically developing readers. Although this pattern has traditionally been interpreted as a compensatory response to left-hemisphere dysfunction, findings from longitudinal, intervention, and network-based studies suggest that compensation alone does not fully account for the diversity of neurobiological findings reported across developmental dyslexia [5]. In particular, evidence demonstrating early right-hemisphere recruitment before formal reading instruction, together with widespread developmental changes in structural and functional connectivity, raises the possibility that right-hemisphere involvement reflects broader processes of reading-network development rather than compensation alone.

Despite these advances, an important conceptual challenge remains unresolved. Across longitudinal, intervention, and neuroimaging studies, similar patterns of early right-hemisphere recruitment have been associated with markedly different developmental outcomes. Some studies report greater right-hemisphere recruitment in individuals who subsequently achieve better reading outcomes or respond successfully to intervention, whereas others describe comparable activation patterns in individuals who show persistent reading difficulties or limited intervention-related improvement. These apparently conflicting findings suggest that similar neural activation patterns may arise through different developmental processes rather than reflecting a single compensatory mechanism. Although existing neurobiological models successfully explain important aspects of brain connectivity, developmental neuroplasticity, and reading acquisition, they generally address these mechanisms separately and therefore provide only a partial explanation for the heterogeneous developmental patterns reported across the literature. This unresolved heterogeneity provides the conceptual motivation for the integrative framework proposed in the present review.

The aim of this narrative review is to integrate evidence from developmental neurobiology, network neuroscience, longitudinal neuroimaging, and intervention research to examine the developmental mechanisms underlying early right-hemisphere recruitment in developmental dyslexia. Rather than proposing a new neurobiological mechanism, this review develops an integrative developmental framework that synthesizes existing evidence into a coherent account of reading-network reorganization. Specifically, the proposed Developmental Neuroplasticity Framework for Reading Network Reorganization explains how interactions among early neurodevelopmental vulnerability, distributed network connectivity, developmental neuroplasticity, and environmental experience may give rise to multiple developmental trajectories of reading-network organization. Within this framework, early right-hemisphere recruitment is conceptualized as one possible adaptive developmental outcome rather than as a universal compensatory response to left-hemisphere dysfunction. By integrating findings that have largely been discussed within separate theoretical perspectives, the framework provides a developmental explanation for the heterogeneous neurobiological patterns reported across individuals with developmental dyslexia while generating empirically testable predictions for future longitudinal investigations of reading-network development.

### Literature Search Strategy

This article presents a narrative review that integrates current evidence on developmental neuroplasticity and reading-network reorganization in developmental dyslexia in order to develop a conceptual framework explaining heterogeneous developmental trajectories. The literature was identified through comprehensive searches conducted via the University of Haifa Library academic search system, with searches performed across PubMed, Scopus, Web of Science, and Google Scholar. Searches were conducted using combinations of keywords including developmental dyslexia, reading network, neuroplasticity, right hemisphere, brain connectivity, white matter, functional MRI, diffusion tensor imaging, reading intervention, longitudinal studies, and related terms. Reference lists of relevant articles were also screened to identify additional publications.

Priority was given to peer-reviewed empirical studies, longitudinal neuroimaging investigations, intervention studies, systematic reviews, meta-analyses, and influential theoretical papers that directly addressed developmental changes in reading-related brain networks, neural plasticity, and compensatory mechanisms in developmental dyslexia. Particular emphasis was placed on recent literature and we also included seminal studies that have substantially shaped current theoretical understanding of developmental dyslexia and reading-network organization.

As a narrative review, this article does not follow a formal systematic review protocol or meta-analytic methodology. Instead, the selected literature was critically synthesized to integrate existing evidence and support the development of the proposed conceptual framework. The literature search was updated prior to manuscript revision to incorporate recently published studies relevant to the proposed framework.

## 2. Current Neurobiological Models of Developmental Dyslexia

Developmental dyslexia has traditionally been explained by localization-based neurobiological models that emphasize dysfunction within a predominantly left-hemisphere reading network, particularly the temporo-parietal, occipito-temporal, and inferior frontal regions [6]. For more than two decades, these models have provided the dominant neurobiological account of dyslexia by highlighting the specialized role of left-hemisphere language regions in reading. Within this framework, the left temporo-parietal region is considered central to phonological decoding, and disruption of this system represents one of the most consistently reported neurobiological features of developmental dyslexia [7]. Likewise, reduced activation of the Visual Word Form Area (VWFA) has been consistently identified as a characteristic neural marker of dyslexia [6,8].

More recently, advances in neuroimaging have shifted the field from localization-based accounts toward network-oriented perspectives, conceptualizing reading as the coordinated activity of multiple cortical regions linked by white-matter pathways rather than as the function of isolated brain regions or hemispheric specialization alone. Core components of this large-scale reading network include the left superior temporal gyrus, inferior frontal gyrus, fusiform gyrus, and the white-matter pathways connecting these regions [9,10]. Accordingly, contemporary neurobiological models increasingly characterize developmental dyslexia as involving alterations in neural connectivity in addition to regional dysfunction, emphasizing interactions across distributed neural systems rather than abnormalities confined to individual cortical regions [10].

These developments have substantially advanced current understanding of the neurobiology of developmental dyslexia by demonstrating that reading depends on the maturation and integration of distributed neural systems rather than isolated cortical regions. However, existing models primarily emphasize individual aspects of reading development, including regional specialization, network connectivity, or neuroplasticity, and therefore provide only a partial explanation for the heterogeneous patterns of early right-hemisphere recruitment reported across longitudinal, intervention, and neuroimaging studies. This limitation does not diminish the value of these models; rather, it highlights the need for an integrative developmental perspective that considers how these complementary mechanisms interact across development to produce multiple trajectories of reading-network organization. The following section therefore outlines the neurodevelopment of the typical reading network before examining how this developmental trajectory diverges in children with developmental dyslexia.

## 3. The Neurodevelopment of the Typical Reading Network

Reading acquisition builds upon the development of oral language, which provides the linguistic foundation for literacy [1]. As spoken language matures, the neural systems supporting language establish the biological substrate upon which the reading network gradually develops during literacy acquisition. Reflecting this distributed neurobiological architecture, reading relies on coordinated activity across a network comprising the left superior temporal gyrus, inferior frontal gyrus, fusiform gyrus, and the white-matter pathways connecting these regions [9]. Consequently, successful reading depends on the integration of dorsal and ventral processing pathways rather than on the activity of isolated cortical regions, underscoring the network-based organization of the developing reading system [11].

Typical literacy development is accompanied by a gradual transition from relatively greater right-hemisphere involvement toward increasing left-hemisphere specialization for language and reading [5]. Importantly, both hemispheres contribute to language development throughout early literacy acquisition, although functional specialization progressively shifts toward the left hemisphere as reading proficiency increases [12]. Consistent with this developmental progression, language lateralization evolves from relatively diffuse hemispheric representation during infancy to increasingly left-lateralized organization in adulthood [13]. Rather than reflecting a fixed sequence, this developmental transition is shaped by continuous interactions between brain maturation, experience, and neuroplasticity.

The Visual Word Form Area (VWFA) acquires its functional specialization through structural and functional connections with the broader language network rather than through intrinsic regional properties alone [14]. This specialization is thought to be constrained by connectivity patterns established before reading begins, with the functional selectivity of the VWFA emerging from pre-existing connections to language-related regions [15]. During the early stages of literacy acquisition, the VWFA undergoes rapid functional refinement as orthographic representations become progressively more specialized [16].

Consistent with this developmental sequence, the dorsal reading pathway is particularly engaged during the early stages of reading acquisition, when children rely heavily on effortful letter-by-letter decoding [17]. As reading skills become more fluent, processing gradually shifts toward the ventral pathway, reflecting the increasing efficiency of visual word recognition and automatic orthographic processing [17]. These developmental changes illustrate how functional specialization emerges through progressive reorganization of distributed neural networks rather than through maturation of isolated cortical regions.

Together, these findings indicate that typical reading development reflects one normative developmental trajectory through which distributed neural systems progressively specialize for efficient reading. This trajectory emerges through continuous interactions among brain maturation, structural and functional connectivity, neuroplasticity, and reading experience. Understanding this normative developmental process provides an essential foundation for interpreting how alternative developmental trajectories may emerge in developmental dyslexia. The following section therefore examines how atypical neurodevelopment alters the maturation and organization of the reading network.

## 4. Developmental Dyslexia as an Atypical Trajectory of Reading Network Development

Developmental dyslexia is increasingly recognized as a neurodevelopmental disorder in which atypical brain organization emerges before formal reading instruction and continues to evolve throughout literacy acquisition. Longitudinal and prospective neuroimaging studies have identified structural and functional differences in children at familial risk for dyslexia prior to learning to read, suggesting that alterations in the developing reading system precede the onset of reading difficulties [4]. Supporting this developmental perspective, children who later develop dyslexia may already exhibit less organized white-matter architecture within left temporo-parietal pathways by approximately five years of age, before formal reading instruction begins [3,18].

Rather than reflecting static neural abnormalities, developmental dyslexia is increasingly viewed as an alteration in the developmental trajectory of reading-related neural systems. Longitudinal evidence indicates that the cortical regions and white-matter pathways supporting fluent reading follow different maturational courses in children who subsequently experience reading difficulties, suggesting that dyslexia reflects atypical network development rather than dysfunction confined to isolated cortical regions [2]. These findings support the view that developmental dyslexia represents a divergence from the normative trajectory of reading-network maturation rather than the failure of a single neural system.

These neurodevelopmental differences involve not only cortical regions but also the structural and functional connections that integrate them into a coordinated reading system. White-matter pathways supporting communication among language-related regions appear to develop atypically before the onset of formal literacy, while functional connectivity continues to reorganize throughout reading acquisition. Moreover, reduced connectivity is increasingly considered an early neurodevelopmental marker of vulnerability rather than merely a consequence of poor reading performance [3,19]. Importantly, developmental differences in network organization do not necessarily determine a single developmental outcome. Instead, the interaction between early biological vulnerability, ongoing brain maturation, reading experience, and neuroplastic adaptation may influence how reading networks continue to reorganize throughout childhood.

Viewed together, these findings indicate that developmental dyslexia is characterized by atypical maturation of distributed reading networks rather than localized cortical dysfunction. Equally importantly, these developmental alterations should not be regarded as fixed outcomes but as evolving processes that remain responsive to experience-dependent neuroplasticity [14]. Consequently, children with similar early neurobiological characteristics may follow different developmental trajectories depending on how these interacting biological and environmental influences shape subsequent network organization.

Taken together, current evidence suggests that developmental dyslexia is best understood as a dynamic developmental process involving multiple potential trajectories of reading-network maturation rather than a single neurobiological pathway. This developmental perspective provides the foundation for examining how neuroplastic mechanisms contribute to adaptive and maladaptive reorganization of reading networks throughout literacy acquisition. The following section therefore examines the role of developmental neuroplasticity in shaping these evolving neural pathways.

## 5. Developmental Neuroplasticity and Network Reorganization

Reading acquisition is characterized by remarkable developmental neuroplasticity throughout childhood, enabling the neural systems that support literacy to reorganize in response to brain maturation, reading experience, and educational instruction [4]. Neuroimaging studies indicate that these neural systems remain highly responsive to environmental input and literacy experience, demonstrating that the biological architecture underlying reading continues to mature well beyond the onset of formal reading instruction [14].

Evidence from intervention studies further demonstrates that the developing reading system remains highly adaptable. Successful intervention has been associated with widespread functional reorganization, including increased activation within left-hemisphere language regions accompanied by changes extending across distributed neural systems [20,21]. In addition, structural connectivity within the reading network has been shown to be closely associated with reading ability, further highlighting the importance of white-matter organization for efficient reading development [22]. Together, these findings indicate that neuroplasticity operates at the level of distributed reading networks rather than isolated cortical regions.

Developmental plasticity is also reflected in the structural architecture of the reading network. Reading intervention has been associated with changes in white-matter organization and functional connectivity, indicating that experience-dependent plasticity encompasses both structural and functional remodeling of the neural systems underlying reading [23,24]. Likewise, intervention has been shown to strengthen neural connections and partially normalize patterns of connectivity, suggesting that its effects extend beyond regional activation to encompass large-scale network reorganization [25]. Notably, experience-dependent changes in white-matter properties have been observed within only a few weeks of intervention, illustrating that structural plasticity can emerge rapidly during childhood [24]. Consistent with these observations, evidence-based literacy interventions have been associated with improvements in behavioral performance together with functional and structural brain reorganization [20,21,24,25]. In addition, longitudinal evidence suggests that hemispheric lateralization remains modifiable during literacy development [5]. Collectively, these findings demonstrate that developmental neuroplasticity influences multiple levels of brain organization, including functional activation, structural connectivity, hemispheric lateralization, and large-scale network architecture.

Overall, the available evidence indicates that the neural systems supporting reading retain considerable plasticity throughout childhood. Rather than representing a fixed developmental outcome, the architecture of the reading network continues to respond to experience, instruction, and environmental influences during literacy acquisition [5]. This capacity for adaptive reorganization provides an important biological basis for understanding why children with similar early neurodevelopmental vulnerabilities may follow different developmental trajectories of reading-network organization. It also raises a fundamental question that remains incompletely understood: how is this developmental capacity for network reorganization expressed in the early right-hemisphere recruitment observed in some children with developmental dyslexia? The following section examines the evidence for this phenomenon.

## 6. Evidence for Early Right-Hemisphere Recruitment in Developmental Dyslexia

Accumulating evidence from functional, structural, and longitudinal neuroimaging studies indicates that some individuals with developmental dyslexia recruit right-hemisphere regions more extensively during reading than typically developing readers. Increased activation of right-hemisphere regions during reading-related tasks has been consistently documented in individuals with dyslexia [26]. Likewise, impaired readers exhibit reduced activation within the left-hemisphere reading network accompanied by greater recruitment of right-hemisphere regions during reading and phonological processing tasks [13,27].

Intervention studies further indicate that right-hemisphere recruitment is associated with individual differences in reading improvement. Increased activation of the right inferior frontal gyrus has been linked to gains in phonological processing following intervention [28], while activation of the same region distinguishes children who respond successfully to intervention from those who do not [29]. Similarly, enhanced activation of the right parietal cortex has been associated with improvements in nonword decoding [30]. Consistent with these observations, individuals with dyslexia who showed significant reading gains following intervention also demonstrated greater recruitment of right-hemisphere regions [31]. Collectively, these findings suggest that right-hemisphere recruitment is associated with reading development, although its functional significance appears to vary across individuals and developmental contexts.

Prospective and longitudinal investigations suggest that right-hemisphere recruitment may emerge even before formal reading instruction begins. Children with a familial risk for dyslexia exhibit right-lateralization of the arcuate fasciculus prior to literacy acquisition, whereas typically developing children show predominantly left-lateralized organization [32]. Likewise, preschool children at familial risk demonstrate right-lateralization of white-matter pathways supporting reading, and accelerated maturation of these pathways has been associated with more favorable reading outcomes [31]. Together, these longitudinal findings suggest that stronger right-hemisphere white-matter organization may represent one possible developmental pathway supporting successful reading acquisition in children at familial risk for dyslexia [32].

Overall, evidence from functional neuroimaging, structural imaging, intervention studies, and longitudinal research suggests that early right-hemisphere recruitment is observed in a subset of individuals with developmental dyslexia across multiple developmental stages and experimental paradigms. Although its frequency and functional significance vary across studies, its recurring observation across multiple lines of research has contributed to ongoing interest in understanding its role in reading-network development. At the same time, the available evidence indicates that similar patterns of right-hemisphere recruitment may be associated with different developmental outcomes across studies and individuals. Consequently, the presence of right-hemisphere recruitment alone cannot determine whether it reflects compensation, adaptive neurodevelopment, or another aspect of reading-network reorganization. This unresolved heterogeneity has given rise to several competing interpretations, which are examined in the following section.

## 7. Evidence for Compensatory Neural Recruitment

The previous section summarized evidence demonstrating early right-hemisphere recruitment during reading in individuals with developmental dyslexia. Traditionally, these findings have been interpreted as reflecting compensatory neural recruitment, whereby alternative neural systems are engaged to support reading when the typical left-hemisphere network functions less efficiently. The studies reviewed below constitute the principal empirical basis for this compensatory interpretation.

Support for this interpretation comes from converging findings across functional neuroimaging, intervention, and longitudinal studies, all of which suggest that alternative neural systems may be recruited in response to reduced efficiency within the typical left-hemisphere reading network [33]. Increased activation of right-hemisphere regions during reading has therefore been widely viewed as a compensatory mechanism that helps sustain reading performance in individuals with developmental dyslexia [34]. Similarly, the combination of reduced activation within left-hemisphere speech-processing regions and increased right-hemisphere activation during reading and phonological tasks has frequently been interpreted as evidence of adaptive functional reorganization [13,27].

Intervention studies provide additional support for this perspective. Increased activation of the right inferior frontal gyrus has been associated with improvements in phonological processing following intervention [28], while activation of the same region distinguishes children who respond successfully to intervention from those who do not [29]. Likewise, enhanced activation of the right parietal cortex has been linked to gains in nonword decoding, suggesting that right-hemisphere recruitment may contribute to phonological aspects of reading [30]. Consistent with these findings, individuals with dyslexia who demonstrated significant reading improvement following intervention also showed greater activation of right-hemisphere regions, whereas comparable neural changes were not consistently observed among non-responders [21,29].

Longitudinal evidence has further extended the compensatory account by suggesting that right-hemisphere pathways may contribute to successful reading development even before formal literacy instruction begins. Children with stronger right-hemisphere white-matter organization have been reported to achieve more favorable long-term reading outcomes [34]. Likewise, right-lateralized white-matter pathways have been proposed to support either compensatory or alternative developmental routes to reading acquisition in children at familial risk for dyslexia [32]. In addition, right-hemispheric pathways have been described as potential protective mechanisms that facilitate successful reading development in at-risk children [31]. Supporting this view, longitudinal neuroimaging studies have shown that children with familial risk exhibit right-lateralization of the arcuate fasciculus before formal reading instruction, whereas typically developing children display predominantly left-lateralized organization [4].

Taken together, these findings provide substantial support for the view that right-hemisphere recruitment can contribute to successful reading performance under at least some developmental conditions. At the same time, the evidence also reveals important conceptual challenges. Several observations—including the presence of right-hemisphere recruitment before formal reading instruction, variability in intervention response, and considerable heterogeneity in developmental outcomes—are not fully explained by a compensation-based interpretation alone. Consequently, although the compensatory account remains an important and well-supported explanation, it may not fully capture the range of developmental processes reflected in early right-hemisphere recruitment. The following section therefore examines competing interpretations that seek to explain this empirical heterogeneity.

## 8. Competing Interpretations of Early Right-Hemisphere Recruitment

The previous section reviewed evidence supporting the interpretation that early right-hemisphere recruitment may contribute to reading development in individuals with developmental dyslexia. The evidence reviewed thus far clearly demonstrates that early right-hemisphere recruitment is a reproducible feature observed in at least a subset of individuals with developmental dyslexia. The central question, therefore, is no longer whether this phenomenon occurs, but how its functional significance should be interpreted. Although compensatory recruitment remains the dominant explanation, findings from functional neuroimaging, structural imaging, longitudinal studies, and intervention research indicate that similar patterns of right-hemisphere recruitment may be associated with different developmental outcomes. This empirical heterogeneity suggests that no single explanatory model fully accounts for the diversity of neurobiological findings reported across developmental dyslexia [35].

One major source of debate concerns the traditional distinction between normalization and compensation. Perdue et al. [36] argue that this distinction represents an oversimplified dichotomy and propose that a dynamic network-based interpretation provides a more appropriate framework for understanding intervention-related neural changes. Supporting this view, distinct patterns of resting-state functional connectivity have been associated with different remediation outcomes, suggesting that children may follow multiple neurodevelopmental pathways rather than a single compensatory trajectory [37]. Likewise, successful intervention has been associated with reorganization across distributed neural systems rather than isolated changes within individual cortical regions [21]. Together, these findings suggest that similar patterns of right-hemisphere recruitment may reflect different neurodevelopmental pathways depending on the broader developmental context rather than a single compensatory mechanism.

Additional findings indicate that increased right-hemisphere activation cannot always be interpreted as evidence of successful compensation. Children who failed to improve following intervention also demonstrated increased activation in the right superior temporal and bilateral inferior frontal regions [38]. Likewise, inferior frontal activation has been interpreted as reflecting compensatory processing, inefficient neural processing, or task-dependent recruitment depending on the experimental paradigm [39,40]. Furthermore, evidence suggests that the neural profile of developmental dyslexia changes across development, making it important to distinguish chronological age, reading age, and intervention history when interpreting neuroimaging findings [41]. Collectively, these findings indicate that interpretation of right-hemisphere recruitment requires consideration of developmental stage, intervention history, and task demands rather than neural activation patterns alone.

Alternative developmental explanations have also been proposed. Di Pietro et al. [14] report that current evidence cannot determine whether altered connectivity represents a cause or a consequence of reading impairment. They further propose that some connectivity alterations may reflect delayed maturation, whereas others may arise from reduced reading experience, suggesting that disorder-related and experience-dependent mechanisms coexist within the developing reading network. Similarly, Helland [5] notes that the relationship between atypical hemispheric lateralization and developmental dyslexia remains debated, including whether altered lateralization represents a cause or a consequence of reading difficulties [5,35]. Together, these observations further support the view that multiple biological and experiential mechanisms may contribute to similar patterns of network organization across development.

Collectively, current evidence indicates that no single interpretation fully explains early right-hemisphere recruitment in developmental dyslexia. Rather than supporting a single developmental mechanism, the available findings suggest that similar patterns of neural organization may emerge through multiple interacting developmental processes that vary across individuals and developmental stages. Consequently, understanding early right-hemisphere recruitment requires an integrative developmental perspective that considers how neurodevelopmental vulnerability, brain connectivity, developmental neuroplasticity, and environmental experience interact over time to shape reading-network organization. The following section therefore reviews the biological mechanisms that may underlie these interacting developmental processes.

## 9. Biological Mechanisms Supporting Early Right-Hemisphere Recruitment

The previous section examined competing interpretations of early right-hemisphere recruitment in developmental dyslexia. A growing body of evidence suggests that this phenomenon is unlikely to be explained by a single neurobiological process. Instead, several interacting mechanisms have been proposed, including alterations in white-matter maturation, large-scale neural connectivity, interhemispheric communication, and audiovisual integration [31]. Collectively, these mechanisms provide a biological context for understanding how reading networks may reorganize across development rather than identifying a single cause of atypical reading outcomes. The following sections review the principal biological mechanisms discussed in the current literature.

Abnormal maturation of white-matter pathways supporting communication within the reading network represents one of the most consistently reported neurobiological features of developmental dyslexia. In particular, abnormalities of the arcuate fasciculus have been repeatedly associated with phonological deficits and impaired reading development [42]. Longitudinal studies further demonstrate that children with a familial risk for dyslexia exhibit greater right-lateralization of reading-related white-matter pathways before formal reading instruction than children without such risk [32]. Likewise, stronger right-hemisphere white-matter organization has been associated with more favorable long-term reading outcomes, suggesting that individual differences in structural maturation may influence developmental reading trajectories [34].

Beyond white-matter development, increasing evidence indicates that developmental dyslexia involves altered organization of large-scale structural and functional brain networks. The concept of developmental dyslexia as a disorder of distributed brain connectivity can be traced to the classical notion of a disconnection syndrome [43] and is supported by contemporary evidence demonstrating widespread alterations in structural connectivity across distributed reading networks [44]. Functional connectivity studies likewise demonstrate altered communication between phonological processing regions in the inferior frontal cortex and temporal language areas, supporting the view that dyslexia reflects disrupted network communication rather than dysfunction confined to isolated cortical regions [45]. Furthermore, disrupted interhemispheric communication between the right auditory cortex and left Broca’s area has also been reported, further emphasizing the importance of large-scale connectivity in the neurobiology of developmental dyslexia [46]. Together, these findings indicate that atypical reading development is best understood as a disturbance of distributed neural communication rather than localized cortical dysfunction.

Interhemispheric communication has also been proposed as an important mechanism underlying atypical developmental trajectories. Differences in corpus callosum maturation may influence hemispheric lateralization during reading acquisition [5]. Likewise, Yu et al. [31] summarize evidence suggesting that genetic susceptibility may contribute to atypical corpus callosum development, thereby providing a structural basis for enhanced interhemispheric connectivity. Such connectivity has consequently been proposed to facilitate greater engagement of right-hemisphere language systems in children at familial risk for dyslexia [31].

Another mechanism involves audiovisual integration during reading acquisition. Fluent reading depends on the efficient integration of visual letter representations with speech sounds, and disruption of this process has been proposed as a core neurobiological mechanism underlying developmental dyslexia [47]. Consistent with this view, impaired integration of orthographic and phonological information may limit the establishment of efficient reading networks during development [2]. Moreover, the integrity of the posterior arcuate fasciculus has been directly associated with audiovisual integration and reading efficiency, providing additional evidence that structural connectivity contributes to the maturation of efficient reading networks [48].

Importantly, these biological mechanisms should not be viewed as independent processes. Rather, they are likely to interact dynamically throughout development, jointly shaping the maturation and reorganization of distributed reading networks during literacy acquisition.

Overall, the available evidence indicates that developmental dyslexia arises from the interaction of multiple neurobiological mechanisms rather than from a single underlying deficit. Alterations in white-matter maturation, large-scale network connectivity, interhemispheric communication, and audiovisual integration appear to operate in concert throughout reading development, although none alone adequately explains the heterogeneity of neuroimaging findings. Taken together, these findings provide the biological rationale for considering early right-hemisphere recruitment within a broader developmental framework of reading-network reorganization. This convergence of evidence provides the biological foundation for the Developmental Neuroplasticity Framework proposed in the following section, which integrates these interacting mechanisms within a unified developmental account of reading-network reorganization.

## 10. A Developmental Neuroplasticity Framework for Reading Network Reorganization in Developmental Dyslexia

The evidence reviewed throughout this article demonstrates that contemporary neurobiological models have substantially advanced our understanding of developmental dyslexia by illuminating complementary aspects of reading-network development. Distributed network models have highlighted the importance of large-scale brain connectivity and white-matter organization [2], developmental neuroplasticity research has demonstrated that reading networks remain dynamically modifiable throughout literacy acquisition [14], and intervention-based models have emphasized experience-dependent functional reorganization together with compensatory neural recruitment [36]. Collectively, these perspectives provide a comprehensive account of many aspects of reading development. However, because they primarily emphasize different components of the developmental process, they do not fully explain why similar patterns of early right-hemisphere recruitment are associated with different developmental outcomes across longitudinal, intervention, and neuroimaging studies [3].

The Developmental Neuroplasticity Framework proposed here addresses this conceptual gap by integrating these complementary perspectives within a unified developmental account of reading-network reorganization. Rather than introducing a new neurobiological mechanism, the framework synthesizes existing evidence to explain how interactions among early neurodevelopmental vulnerability, distributed network connectivity, developmental neuroplasticity, and environmental experience may give rise to multiple developmental trajectories of reading-network organization. Within this framework, early right-hemisphere recruitment is conceptualized as one possible adaptive developmental outcome rather than as a universal compensatory response to left-hemisphere dysfunction. Thus, the proposed framework offers an integrative explanation for the heterogeneity of neurobiological findings while remaining fully consistent with existing empirical evidence. By integrating these complementary perspectives within a developmental framework, the proposed framework provides an interpretive model for understanding why apparently similar patterns of early right-hemisphere recruitment may be associated with different developmental trajectories and reading outcomes across individuals.

From this developmental perspective, developmental dyslexia is conceptualized as a dynamic neurodevelopmental process that begins before formal reading instruction. This view is informed by evidence demonstrating that atypical brain organization, altered white-matter maturation, and differences in distributed network connectivity emerge before reading acquisition and continue to influence literacy development throughout childhood [4]. Rather than assuming a static neural deficit, the framework proposes that these early biological differences create multiple potential developmental pathways through which reading networks continue to mature during literacy acquisition. Which developmental pathway ultimately emerges is likely influenced by ongoing interactions among biological maturation, neuroplasticity, environmental experience, and educational input.

**Figure 1:**
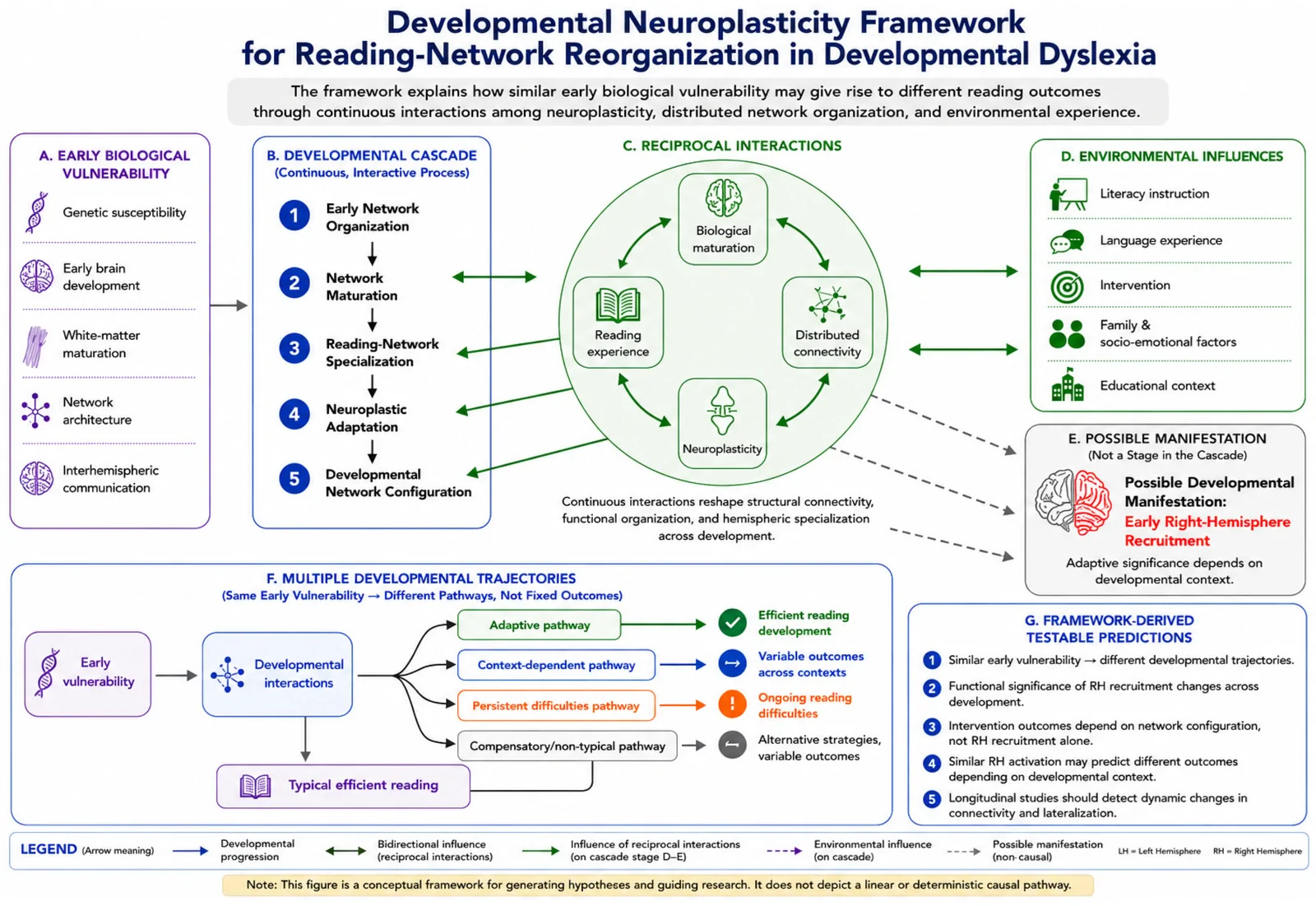
Developmental Neuroplasticity Framework for Reading-Network Reorganization in Developmental Dyslexia.

Reading acquisition is further conceptualized as a process of progressive network specialization driven by reciprocal interactions between biological maturation and reading experience [14]. During typical development, functional specialization gradually becomes increasingly left-lateralized while remaining dependent on coordinated interactions among distributed neural systems [5]. In developmental dyslexia, altered network organization may redirect this developmental process, giving rise to alternative patterns of functional specialization rather than a uniform disruption of isolated cortical regions [3]. Within this framework, right-hemisphere recruitment is therefore viewed as one possible manifestation of adaptive network reorganization whose functional significance depends on the broader developmental context in which it occurs, rather than on neural activation patterns alone. The proposed framework therefore provides an integrative developmental perspective for interpreting why apparently similar patterns of early right-hemisphere recruitment may be associated with different developmental trajectories and reading outcomes, thereby providing a developmental interpretation of the heterogeneous findings reported across longitudinal, intervention, and neuroimaging studies. The proposed Developmental Neuroplasticity Framework is summarized in Figure 1.

The proposed Developmental Neuroplasticity Framework integrates the complementary neurobiological mechanisms reviewed throughout this article into a unified developmental account of reading-network reorganization in developmental dyslexia. Rather than proposing a novel neurobiological mechanism, the framework illustrates how continuous interactions among early biological vulnerability, distributed network organization, developmental neuroplasticity, environmental experience, and educational intervention may jointly shape the developmental reorganization of reading networks across childhood.

The framework depicts a developmental cascade embedded within continuous reciprocal interactions rather than a fixed sequential pathway. The cascade begins with early biological vulnerability, including genetic susceptibility, early brain development, white-matter maturation, network architecture, and interhemispheric communication, and progresses through the initial organization, maturation, specialization, and ongoing reconfiguration of reading-related neural networks during literacy acquisition. Continuous reciprocal interactions among biological maturation, distributed network connectivity, neuroplasticity, and reading experience constitute the central developmental mechanism of the proposed framework, progressively reshaping structural connectivity, functional network organization, and hemispheric specialization throughout development.

Within this framework, early right-hemisphere recruitment is conceptualized as a context-dependent manifestation of developmental reading-network reorganization rather than a universal compensatory response to left-hemisphere dysfunction or a fixed developmental stage. Its functional significance is proposed to depend on the evolving interaction among biological vulnerability, network configuration, neuroplasticity, reading experience, and environmental influences, and may therefore differ across individuals and developmental periods.

The framework further proposes that similar early neurodevelopmental characteristics may give rise to multiple developmental trajectories of reading-network organization. These trajectories may include efficient reading development, adaptive alternative pathways, context-dependent outcomes, or persistent reading difficulties, depending on the dynamic interaction between biological and environmental factors over time. Consequently, similar patterns of right-hemisphere recruitment may predict different developmental outcomes according to the evolving configuration of distributed reading networks rather than reflecting a single compensatory mechanism.

The arrows illustrate proposed developmental progression together with reciprocal interactions, environmental modulation, neuroplastic adaptation, and developmental feedback operating across multiple stages of network organization. Accordingly, the figure should be interpreted as a conceptual developmental framework designed to generate empirically testable hypotheses regarding reading-network development and reorganization, rather than as a fixed, linear, or deterministic causal sequence. Specifically, the framework predicts that comparable early biological vulnerability may lead to different developmental outcomes, that the functional significance of right-hemisphere recruitment changes across development, and that longitudinal studies should detect dynamic changes in structural connectivity, functional organization, and hemispheric lateralization as reading networks continue to reorganize over time.

## 11. Discussion

The principal contribution of the present review is the Developmental Neuroplasticity Framework for Reading-Network Reorganization, which integrates complementary evidence from developmental neurobiology, network neuroscience, longitudinal neuroimaging, and intervention research into a unified developmental account of reading-network organization in developmental dyslexia. Rather than proposing a new neurobiological mechanism, the framework synthesizes existing evidence to explain how interactions among early neurodevelopmental vulnerability, distributed network connectivity, developmental neuroplasticity, and environmental experience may contribute to multiple developmental trajectories of reading-network organization. Within this framework, early right-hemisphere recruitment is conceptualized as one possible manifestation of developmental reading-network reorganization rather than as a universal compensatory response to left-hemisphere dysfunction. Consequently, the principal contribution of the framework is not the introduction of a new biological mechanism but the provision of an integrative developmental explanation for the heterogeneous neurobiological findings reported across developmental dyslexia. By organizing complementary evidence within a single developmental perspective, the framework provides an interpretive model for understanding why apparently similar patterns of early right-hemisphere recruitment may be associated with different developmental trajectories and reading outcomes across individuals.

This developmental perspective shifts emphasis from isolated regional abnormalities toward the dynamic organization and reorganization of distributed reading networks across development. Rather than viewing variability across individuals as evidence against a common neurobiological framework, the proposed model conceptualizes such heterogeneity as an expected consequence of developmental processes operating under different biological and environmental conditions. Within this perspective, reading outcomes emerge through continuous interactions among biological vulnerability, brain maturation, developmental neuroplasticity, reading experience, and environmental influences, giving rise to multiple developmental trajectories. Accordingly, early right-hemisphere recruitment should be interpreted as one possible developmental manifestation of this broader process, whose functional significance depends on the evolving organization of distributed reading networks rather than on hemispheric activation patterns alone.

Beyond providing an integrative account of existing evidence, the proposed framework generates several empirically testable developmental predictions that distinguish it from descriptive models of reading-network development. First, children with similar early neurodevelopmental vulnerability may follow different developmental trajectories depending on differences in developmental neuroplasticity and environmental experience. Second, the functional significance of early right-hemisphere recruitment is expected to evolve across development rather than remain stable over time, reflecting continuous interactions among brain maturation, reading experience, and distributed network reorganization. Third, among children with comparable baseline reading performance, differences in pre-intervention distributed reading-network organization—including measures of structural and functional connectivity—are predicted to contribute to differential responses to comparable evidence-based reading interventions, beyond the presence or absence of early right-hemisphere recruitment alone. Finally, similar patterns of right-hemisphere activation may be associated with different behavioral outcomes depending on developmental stage, network connectivity, and prior reading experience. Collectively, these predictions provide hypotheses that can be evaluated through prospective longitudinal and multimodal neuroimaging studies, thereby allowing the proposed framework to be empirically supported, refined, or challenged.

From this developmental perspective, the framework may also have implications for early identification and intervention. Conceptualizing developmental dyslexia as a dynamic neurodevelopmental process suggests that early differences in reading-network organization should not necessarily be interpreted as fixed neural deficits. Instead, identifying developmental patterns of network organization before formal reading instruction may facilitate earlier identification of children at elevated risk for reading difficulties and contribute to the development of more targeted, developmentally informed intervention strategies. Within the proposed framework, environmental experience may also include emotionally supportive and play-based learning environments that promote motivation, reduce reading-related anxiety, and encourage active engagement in literacy activities. Such experiences may enhance engagement in reading intervention and create conditions that support experience-dependent neuroplasticity, although their specific neurobiological effects remain to be established through future empirical research.

These implications remain conceptual and require empirical validation through prospective longitudinal investigations.

Although the proposed framework primarily focuses on reading-network development during childhood, developmental dyslexia is a lifelong neurodevelopmental condition whose neural organization may continue to evolve beyond formal reading acquisition. From this perspective, the framework may also provide a conceptual basis for understanding individual variability in reading outcomes during adolescence and adulthood and may help inform intervention approaches, educational accommodations, and long-term support strategies across the lifespan. These broader implications remain conceptual and require further empirical investigation.

Another important consideration is the frequent comorbidity between developmental dyslexia and attention-deficit/hyperactivity disorder (ADHD). ADHD is often associated with attentional and executive-function difficulties that may influence reading performance, engagement during reading intervention, and responsiveness to intervention, thereby contributing to variability in reading development among children with reading difficulties [49]. Consequently, comorbid ADHD may also complicate the interpretation of patterns of reading-network reorganization and right-hemisphere recruitment observed across individuals. Although the present framework does not specifically address comorbid neurodevelopmental conditions, future longitudinal studies should consider ADHD comorbidity as a potential moderator when evaluating developmental trajectories of reading-network organization and intervention-related neuroplasticity [49].

The proposed framework should be interpreted in light of several limitations. First, the framework is conceptual and is derived from integrating findings across studies employing diverse neuroimaging methodologies, participant characteristics, languages, and intervention protocols. This methodological heterogeneity limits direct comparison across studies, complicates the interpretation of apparently conflicting findings, and precludes definitive conclusions regarding causal developmental mechanisms. Consequently, the proposed framework should be interpreted as an integrative conceptual model that requires prospective empirical validation. Second, although accumulating evidence supports developmental changes in distributed reading networks, much of the available literature remains cross-sectional, limiting conclusions regarding the temporal dynamics of reading-network reorganization. Finally, although the reviewed evidence supports the hypothesis that early right-hemisphere recruitment may represent one adaptive developmental pathway, current knowledge remains insufficient to determine the developmental conditions under which this trajectory contributes to successful reading outcomes.

Testing the developmental predictions generated by the proposed framework should therefore be a priority for future research. Large-scale longitudinal investigations beginning before formal reading instruction and integrating structural and functional neuroimaging, measures of brain connectivity, and comprehensive assessments of reading development and intervention response will be essential for evaluating the proposed framework. In particular, longitudinal studies examining whether baseline differences in distributed network organization predict later reading outcomes and responsiveness to intervention would provide a direct test of the developmental predictions proposed here. Such studies may help identify neurobiological markers associated with distinct developmental trajectories and clarify how interactions among biological vulnerability, developmental neuroplasticity, environmental experience, and intervention shape reading-network reorganization across development.

In conclusion, the Developmental Neuroplasticity Framework provides an integrative developmental perspective on reading-network reorganization in developmental dyslexia by organizing complementary evidence from developmental neurobiology, network neuroscience, longitudinal neuroimaging, and intervention research within a coherent conceptual model. Rather than advancing a new neurobiological mechanism, the framework proposes an integrative developmental perspective in which interactions among established biological and environmental processes may give rise to multiple developmental trajectories of reading-network organization and may help explain the heterogeneous patterns of early right-hemisphere recruitment reported across the literature. Although the proposed framework requires empirical validation, it provides a coherent developmental perspective for interpreting neurodevelopmental heterogeneity while generating explicit, testable hypotheses that can guide future longitudinal investigations on reading-network development.

## Data Availability

No new data were created or analyzed in this study. Data sharing is not applicable to this article.
